# Dense fine speckled immunofluorescence pattern in a Chinese population: Prevalence and clinical association

**DOI:** 10.1002/jcla.24173

**Published:** 2021-12-24

**Authors:** Keyi Zhang, Zhenzhen Su, Jing Hu, Zhuochun Huang, Chaojun Hu, Bin Yang

**Affiliations:** ^1^ Department of Laboratory Medicine West China Hospital of Sichuan University Chengdu China; ^2^ Department of Rheumatology Peking Union Medical College Hospital Beijing China

**Keywords:** dense fine speckled pattern, exclusion criterion, systemic autoimmune rheumatic diseases

## Abstract

**Objective:**

To provide information on the prevalence and possible clinical association in a Chinese population for medical practice of the dense fine speckled pattern (DFS pattern).

**Methods:**

A retrospective study was conducted with patients who had the DFS pattern from June 2018 to December 2019 in West China Hospital.

**Results:**

A total of 469 patients (1.27% of patients with positive anti‐nuclear antibody indirect immunofluorescence (ANA IIF) test results) revealed the DFS pattern, of which 92.96% had isolated DFS pattern and 23.67% had titers above/equal to 1:320. The average age of patients with the DFS pattern was 43.45 years, and females accounted for 76.97% of them. Ten different kinds of diseases made up the vast majority of the disease spectrum, in which inflammatory or infectious diseases (46.11%), mental diseases (21.45%), and systemic autoimmune rheumatic diseases (SARDs) (18.23%) ranked in the top three. The most common SARDs were rheumatoid arthritis (RA), undifferentiated connective tissue disease (UCTD), and systemic lupus erythematosus (SLE). Forty‐six patients (10.55%) had positive or suspicious extractable nuclear antigen (ENA) antibodies test results and a higher risk of suffering from SARDs. Forty‐seven patients would be missed if the DFS pattern with negative ENA antibodies test result was considered as exclusion criterion of SARDs.

**Conclusions:**

The DFS pattern is basically isolated and with low titer. It is unwise to exclude the diagnosis of SARDs only depending on the appearance of the DFS pattern. Autoimmune diseases‐related antibodies, clinical information of patients, and long‐term follow‐up are of great importance to avoid missed or delayed diagnosis of SARDs.

## Graphical abstract

We found out if the dense fine speckled pattern (DFS pattern) was used as exclusion biomarker of systemic autoimmune rheumatic diseases (SARDs), 68 patients would be missed. Not all SARDs patients had titers above/equal to 1:320 or positive/suspicious extractable nuclear antigen (ENA) antibodies test results. In fact, if lower DFS pattern titers (below 1:320) or DFS patterns with negative ENA antibodies test results were considered as exclusion criteria of SARDs, 36 or 47 patients would be missed. It is unwise to exclude the diagnosis of SARDs only depending on the appearance of the DFS pattern. Autoimmune diseases‐related antibodies, clinical information of patients, and long‐term follow‐up are of great importance to avoid missed or delayed diagnosis of SARDs.

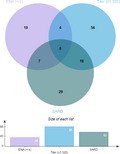

## 1 INTRODUCTION

Autoantibodies directing against human organs, tissues, and cells have been considered as serological hallmarks of various autoimmune diseases.^1^ Among the autoantibodies, anti‐nuclear antibodies (ANAs) play an irreplaceable role in the diagnostic workup of SARDs. The indirect immunofluorescence (IIF) assay based on HEp‐2 cell substrates is extensively used to detect ANA,^2^ and there has been increasing appreciation of the ability of morphological patterns to direct further investigation of specific autoantibodies in recent years,^3^ as reflected in orderly classifying and harmonizing the nomenclature of several relevant HEp‐2 IIF patterns, including the DFS pattern, by The International Consensus on ANA patterns (ICAP).^3^

The DFS pattern, characterized by a dense and heterogeneous speckled staining of both the nucleoplasm of interphase cells and the chromosomal plate of metaphase cells,^4^ was first described in 1994 in interstitial cystitis and later on in a variety of autoimmune conditions, other non‐autoimmune conditions, and even healthy donors.^5^, ^6^ Because sera with the DFS pattern were shown to bind a 70‐kDa protein in immunoblots, the target autoantigen was designated DFS70.^7^

To the best of our knowledge, the DFS pattern/anti‐DFS70 antibodies can be found in a wide spectrum of clinical conditions,^8^, ^9^ but the precise clinical significance of them is still unclear.^10^, ^11^ In addition, due to their low prevalence in SARDs, whether and how can the DFS pattern/anti‐DFS70 antibodies be used to exclude the diagnosis of SARDs remain controversial. Some authors suggested that isolated anti‐DFS70 positivity could be used as exclusion biomarker in SARDs,^6^, ^12^ thus preventing unnecessary further testing, treatment, and distress to patients.^13^ By contrast, other authors claimed that this proposal was difficult to support and found no differences emerged in terms of prevalence of anti‐DFS70 positive samples between SARDs and non‐SARDs groups.^5^, ^14^ Hence, further studies on the DFS pattern/anti‐DFS70 antibodies are required. In this study, we analyzed data on the DFS pattern and investigated its prevalence and possible clinical association in a Chinese population for medical practice of the DFS pattern.

## 2 MATERIALS AND METHODS

### 2.1 Subjects

The study enrolled 115,185 patients who underwent the ANA IIF test in West China Hospital of Sichuan University (one of the largest general teaching hospitals in China with 4300 beds) between June 2018 (The time when our laboratory began to report the DFS pattern to clinicians) and December 2019. SARDs included in the study were RA, systemic lupus erythematosus (SLE), Sjögren's syndrome (SS), scleroderma, dermatomyositis (DM), systemic vasculitis, and undifferentiated connective tissue disease (UCTD).

### 2.2 ANA IIF tests and criteria for determination of the DFS pattern

ANA tests were measured in sera by IIF substrated with HEp‐2 cells (Euroimmun, Germany), using serial dilutions commencing at 1:100.^2^ Slides were read by two qualified and experienced technologists, and would be read by a third one if they did not reach an agreement. The DFS pattern should be carefully distinguished from other patterns such as the homogeneous pattern or the fine speckled pattern.

**FIGURE 1:**
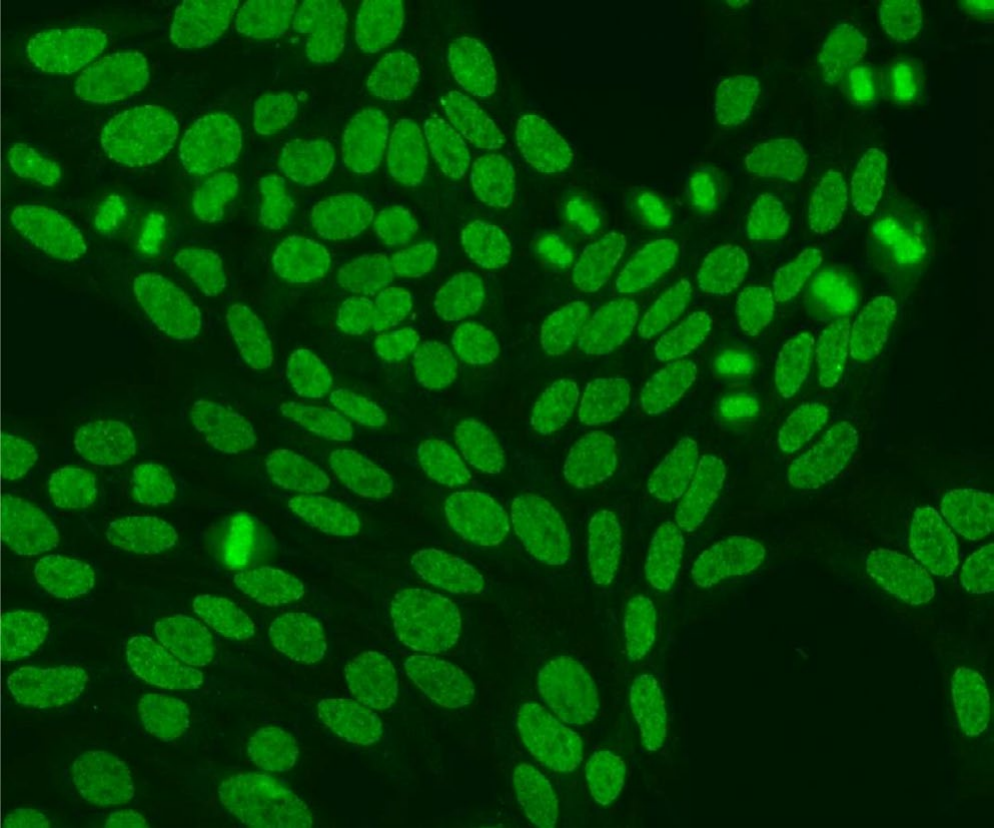
The dense fine speckled pattern (DFS pattern)

The DFS pattern was characterized by three morphologic features^8^, ^15^: (1) Fine speckles distributed throughout the interphase nucleus (sparing the nucleoli) with characteristic heterogeneity in their size, brightness, and distribution; (2) denser and looser areas of speckles throughout the interphase nucleus; and (3) strong speckled pattern in the metaphase plate (maintaining the typical granularity), with some coarse speckles standing out. The DFS pattern is shown in Figure 1.

### 2.3 Detection of other autoantibodies

Extractable nuclear antigen (ENA) autoantibodies, including anti‐U1RNP, Sm, SSA (Ro52 was measured as part of SSA), SSB, Scl‐70, Jo‐1, and Rib antibodies, were evaluated by line immunoblot assay (LIA) via EUROBlotOne (Euroimmun, Germany).^2^ Anti‐double‐stranded DNA (anti‐dsDNA), antikeratin antibodies (AKA), and anti‐neutrophil cytoplasmic antibodies (ANCA) were detected by IIF (Euroimmun, Germany), using serial dilutions commencing at 1:10.^2^ Anti‐cyclic citrullinated peptide (CCP) antibodies were tested on e601 (Roche Diagnostics, Germany), while rheumatoid factors (RFs) were measured by IMMAGE 800 (Beckman Coulter, America).

### 2.4 Other information

Other clinical and laboratory information such as demographic characteristics, diagnosis, and clinical serum index results was collected from the hospital information system and the hospital laboratory information system.

### 2.5 Ethical statements

This study has gained the ethical approval and consent of West China Hospital Ethics Committee. As a retrospective analysis of routinely collected programmatic data, all patient information was de‐identified and precluded the requirement of informed consent.

### 2.6 Data analysis

The statistical software SPSS 19.0 was used for statistical analysis. K‐S test was used to judge whether the results were normally distributed. The continuous variables satisfying the normal distribution were expressed as “mean ± standard deviation,” otherwise as “median (interquartile interval).” For quantitative data, *t*‐test or variance analysis was performed if the data were in line with normal distribution and even variance, otherwise nonparametric test was used. Chi‐square test or Fisher's exact test was used for counting data. The threshold for statistical significance was set at *p* = 0.05. The bar chart was made by Origin 2018. The Venn diagram was made on the website (http://www.ehbio.com/test/venn/#/).

## 3 RESULTS

### 3.1 Main characteristics of the study population

**FIGURE 2:**
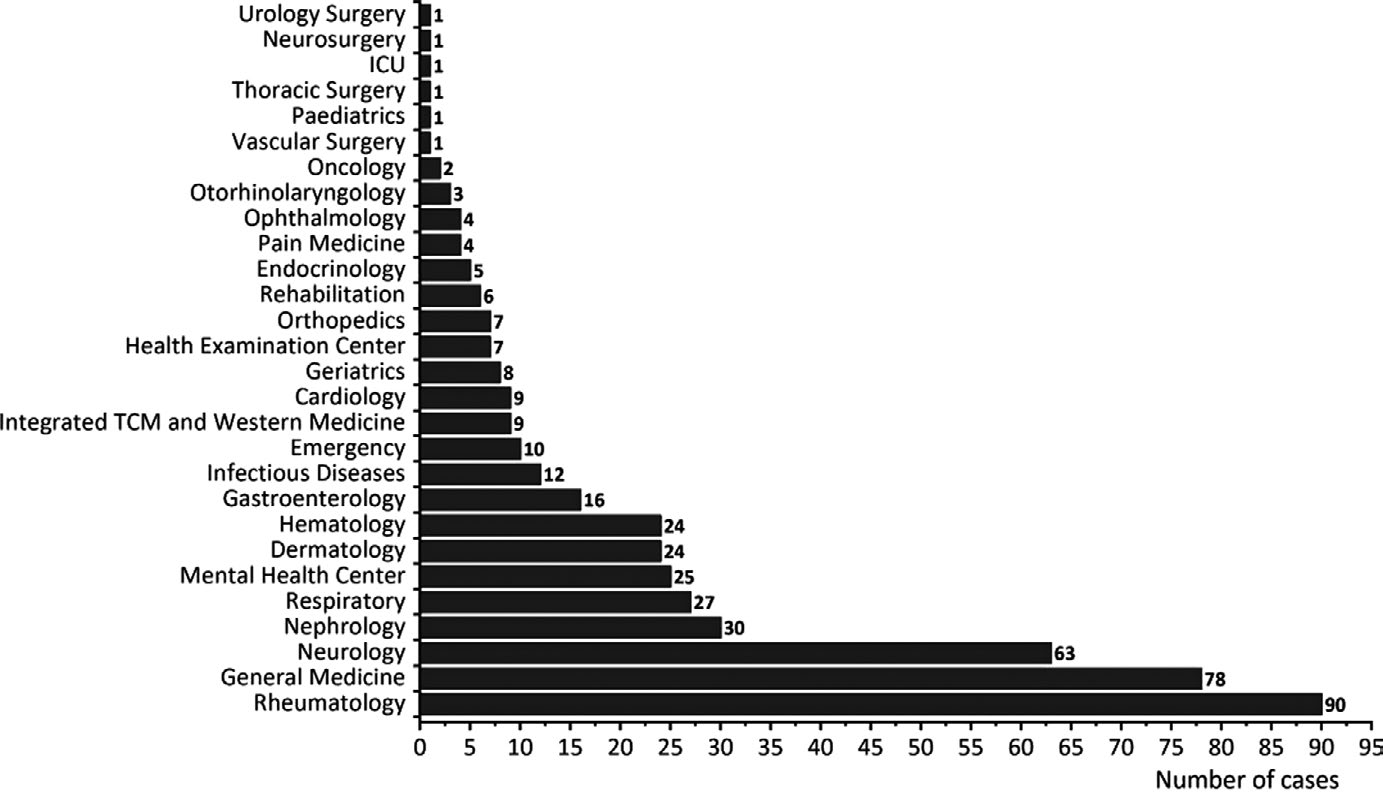
Department distribution of patients with the dense fine speckled pattern (DFS pattern)

A total of 115,185 patients who underwent the ANA IIF test from June 2018 to December 2019 were enrolled in our study, of which 36,869 had positive ANA IIF test results (32.01%), 469 revealed the DFS patterns (1.27% of patients with positive ANA IIF test results), and 111 patients (23.67%) had DFS pattern titers above/equal to 1:320. The average age of patients with the DFS pattern was 43.45 years, and females accounted for 76.97% of them. Among the 28 different departments from which these 469 patients came, rheumatology (19.19%), general medicine (16.63%), and neurology (13.43%) ranked in the top three, as shown in Figure 2. The overwhelming majority of patients had isolated DFS patterns (436 cases, 92.96% of patients with the DFS pattern), nevertheless, other patterns were also identified in 33 patients (7.04% of patients with the DFS pattern), including the nucleolar pattern, nuclear dots pattern, cytoplasmic pattern, etc. Forty‐six patients had positive or suspicious ENA antibodies test results (10.55% of 436 patients who had both the DFS pattern and ENA antibodies test results). Additionally, anti‐dsDNA, AKA, ANCA, CCP antibodies and RF were found in 2 (0.52% of 381 patients who had both the DFS pattern and anti‐dsDNA results), 19 (positive or suspicious, 31.67% of 60 patients who had both the DFS pattern and AKA test results), 14 (suspicious, 8.97% of 156 patients who had both the DFS pattern and ANCA test results), 36 (34.95% of 103 patients who had both the DFS pattern and CCP test results), and 41 (11.39% of 360 patients who had both the DFS pattern and RF test results), respectively.

### 3.2 Relationship among disease spectrum, ENA antibodies test results, and titer of the DFS pattern

**FIGURE 3:**
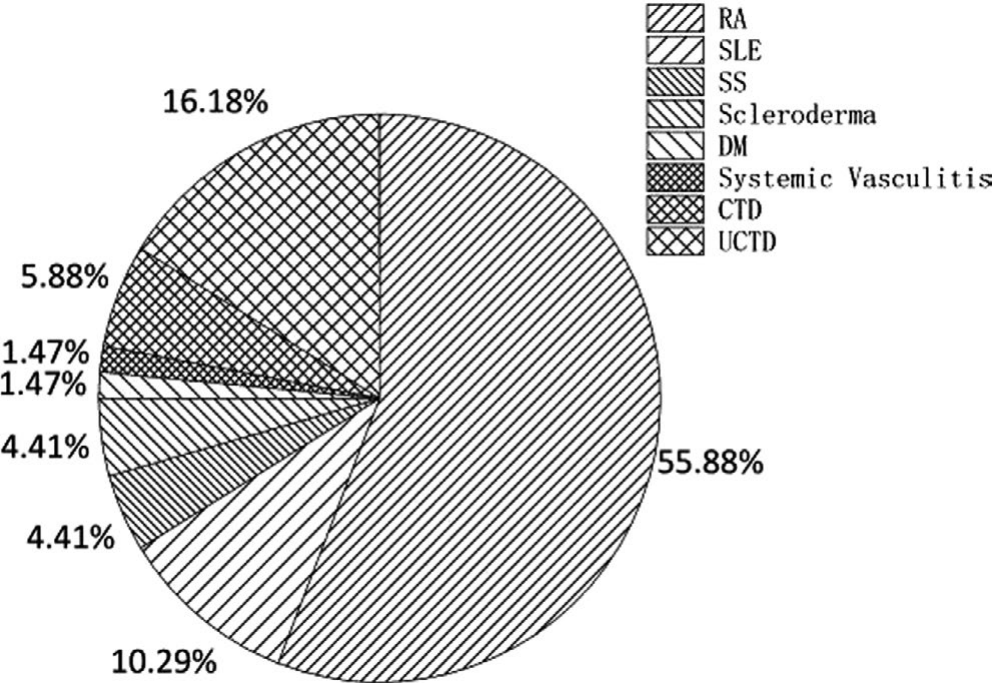
The composition of systemic autoimmune rheumatic diseases (SARDs)

We investigated the relationship of the DFS pattern with diseases. In all patients with DFS patterns, 373 had definitive clinical diagnosis (79.53%). Ten different kinds of diseases (as shown in Table 1) made up the vast majority of the disease spectrum, in which inflammatory or infectious diseases, mental diseases, and SARDs ranked in the top three, with proportions of 46.11%, 21.45%, and 18.23%, respectively. Figure 3 indicates the composition of SARDs. The most common SARDs in patients with DFS patterns were RA (55.88% of the 373 patients), UCTD (16.18% of the 373 patients), and SLE (10.29% of the 373 patients). Then, we made a comparison of the disease spectrum of patients with different ENA antibodies results and DFS pattern titers, finding that patients with positive/suspicious ENA antibodies test results (*p* = 0.001) or titers above/equal to 1:320 (*p* = 0.001) had a higher risk of suffering from SARDs. Besides, it seems that patients with titers above/equal to 1:320 were more likely to have hypertension (*p* = 0.04).

**TABLE 1:** Diseases of patients with different extractable nuclear antigen (ENA) antibodies and dense fine speckled pattern (DFS pattern) titers

	Total (*n* = 373)*	ENA (*n* = 347)**		*p*	Titer (*n* = 373)*		*p*
		Positive/suspicious (*n* = 38)	Negative (*n* = 309)		>or = 1:320 (*n* = 93)	<1:320 (*n* = 280)	
Diseases							
SARD	68 (18.23%)	15 (39.47%)	47 (15.21%)	0.001	28 (30.11%)	40 (14.29%)	0.001
Organ‐specific autoimmune diseases	12 (3.22%)	3 (7.89%)	8 (2.59%)	0.108	3 (3.23%)	9 (3.57%)	1.000
Abnormal pregnancy	12 (3.22%)	3 (7.89%)	6 (1.94%)	0.064	4 (4.30%)	6 (2.14%)	0.275
Mental diseases	80 (21.45%)	6 (15.79%)	71 (22.98%)	0.409	14 (15.05%)	66 (23.57%)	0.108
Neurological diseases	11 (2.95%)	1 (2.63%)	10 (3.24%)	1.000	3 (3.23%)	8 (2.86%)	1.000
Neoplasm	20 (5.36%)	3 (7.89%)	14 (4.53%)	0.414	4 (4.30%)	16 (5.71%)	0.792
Inflammatory or infectious diseases	172 (46.11%)	16 (42.11%)	146 (47.25%)	0.607	46 (49.46%)	126 (45.00%)	0.473
Hypertension	54 (14.48%)	7 (18.42%)	43 (13.92%)	0.463	20 (21.51%)	34 (12.14%)	0.040
Diabetes mellitus	35 (9.38%)	5 (13.16%)	29 (9.39%)	0.398	11 (11.83%)	24 (8.57%)	0.411
Coronary heart disease/Atherosclerosis	26 (6.97%)	3 (7.89%)	23 (7.44%)	1.000	10 (10.75%)	16 (5.71%)	0.104

Note

The pie graph in the top‐left corner indicates the composition of systemic autoimmune rheumatic disease (SARD).

* 373 patients had definite diagnosis.

** 347 patients had definite diagnosis and underwent extractable nuclear antigen (ENA) antibodies test as well.

**FIGURE 4:**
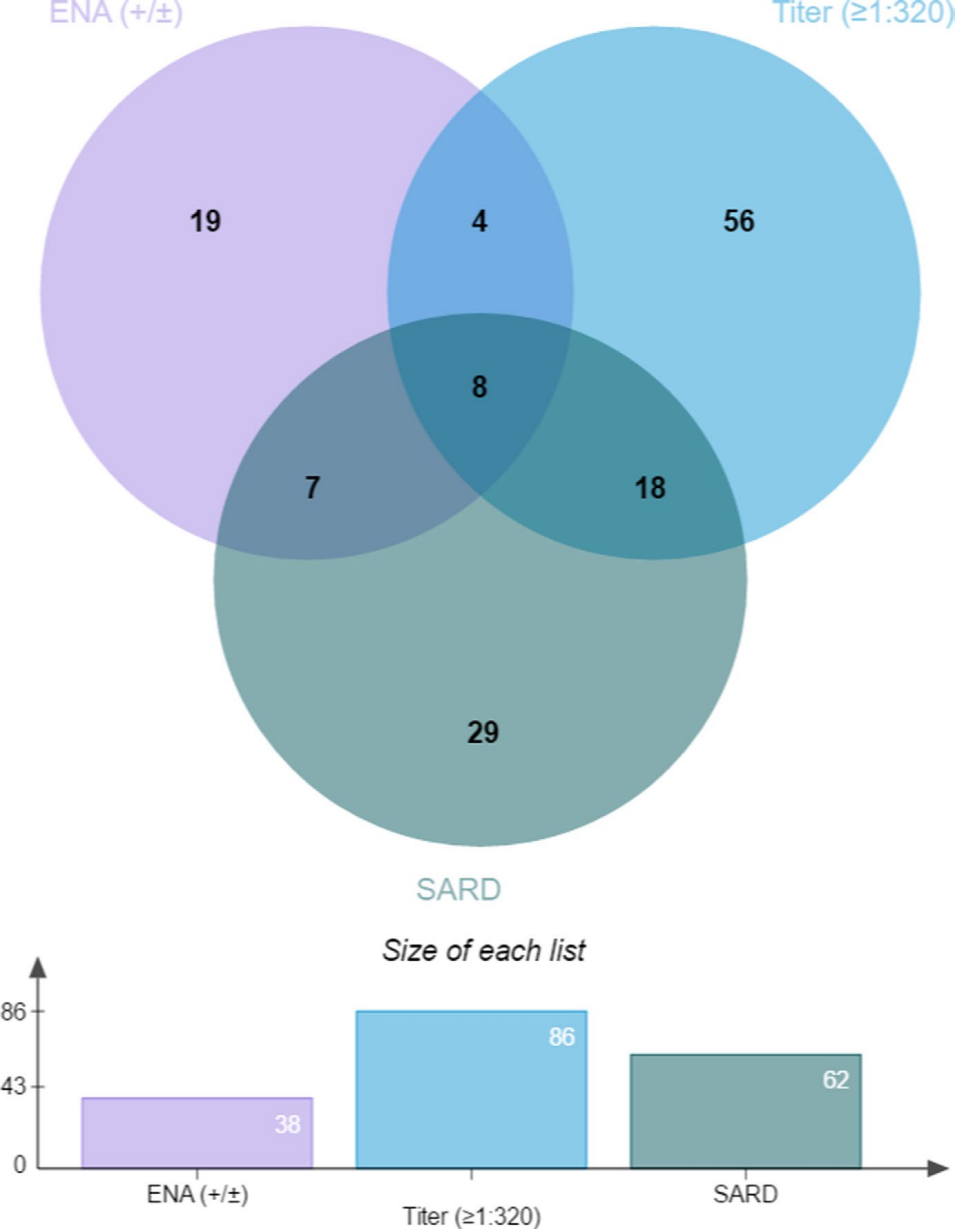
Relationship among systemic autoimmune rheumatic disease (SARD), extractable nuclear antigen (ENA) antibodies, and titers in patients with dense fine speckled patterns (DFS patterns). (All patients included in the Venn diagram had extractable nuclear antigen (ENA) antibodies test results)

In order to illustrate the relationship among DFS pattern titers, SARDs, and ENA antibodies test results in patients with DFS patterns visually, we created a Venn diagram. As shown in Figure 4, the blue, gray‐green, and purple circles represented patients with titers above/equal to 1:320, SARDs, and positive/suspicious ENA antibodies test results, respectively. Interestingly, we found out that not all SARDs patients had titers above/equal to 1:320 or positive/suspicious ENA antibodies test results. In fact, if lower DFS pattern titers (below 1:320) or DFS patterns with negative ENA antibodies test results were considered as exclusion criteria of SARDs, 36 or 47 patients would be missed.

### 3.3 Characteristics of patients with isolated and complex DFS patterns

We divided the 469 patients into two groups: isolated DFS pattern and complex DFS patterns groups. For clarification, isolated DFS pattern represented that patients only had DFS patterns in their ANA IIF test results, while complex DFS patterns indicated that other patterns were also identified. Inflammatory or infectious diseases, mental diseases, and SARDs ranked in the top three, no matter in which group. In isolated DFS pattern group and complex DFS patterns group, RA was responsible for more than half of all the SARDs. Other SARDs including SLE, SS, etc. were also found, but there was no significant difference in the prevalence of SARDs between these two groups. As for ENA antibodies, anti‐SSA antibodies (5.73%) were the most common antibodies, followed by anti‐U1RNP (2.06%) and anti‐Scl‐70 antibodies (1.83%). Almost the same situation was found in isolated and complex DFS patterns groups, and there was no significant difference in the prevalence of ENA antibodies between two groups. We also detected other antibodies (such as anti‐DNA, AKA, etc.) and clinical serum indexes reflecting blood routine parameters, liver, renal and immunologic function, but unfortunately, no significant difference appeared between two groups except for gamma‐glutamyl transferase (GGT) (*p* = 0.049). Detailed characteristics of these patients are shown in Table 2.

**TABLE 2:** Characteristics of patients with isolated and complex dense fine speckled patterns (DFS patterns)

	Total (*n* = 373)*	Isolated DFS pattern (*n* = 345)	Complex DFS patterns (*n* = 28)	*p*
Diseases				
SARD	68 (18.23%)	59 (17.10%)	9 (32.14%)	0.070
Organ‐specific autoimmune diseases	12 (3.22%)	10 (2.90%)	2 (7.14%)	0.225
Abnormal pregnancy	12 (3.22%)	12 (3.48%)	0 (0%)	–
Mental diseases	80 (21.45%)	75 (21.74%)	5 (17.86%)	0.812
Neurological diseases	11 (2.95%)	11 (3.19%)	0 (0%)	–
Neoplasm	20 (5.36%)	19 (5.51%)	1 (3.57%)	1.000
Inflammatory or infectious diseases	172 (46.11%)	162 (46.96%)	10 (35.71%)	0.325
Hypertension	54 (14.48%)	52 (15.07%)	2 (7.14%)	0.401
Diabetes mellitus	35 (9.38%)	33 (9.57%)	2 (7.14%)	1.000
Coronary heart disease/Atherosclerosis	26 (6.97%)	25 (7.25%)	1 (3.57%)	0.708
ENA antibodies (positive/ suspicious)	52 (11.93%, *n* = 436)**	46 (11.36%, *n* = 405)	6 (19.35%, *n* = 31)	0.243
anti‐U1RNP	9 (2.06%)	8 (1.98%)	1 (3.23%)	0.488
anti‐Sm	3 (0.69%)	3 (0.74%)	0 (0%)	–
anti‐SSA	25 (5.73%)	23 (5.68%)	2 (6.45%)	0.695
anti‐ SSB	2 (0.46%)	2 (0.49%)	0 (0%)	–
anti‐Scl−70	8 (1.83%)	6 (1.48%)	2 (6.45%)	0.105
anti‐Jo−1	3 (0.69%)	3 (0.74%)	0 (0%)	–
anti‐Rib	2 (0.46%)	1 (0.25%)	1 (3.23%)	0.137
Other antibodies				
anti‐dsDNA	2 (0.52%, *n* = 381)**	2 (0.57%, *n* = 353)	0 (0%, *n* = 28)	–
AKA	19 (31.67%, *n* = 60)**	17 (29.31%, *n* = 58)	2 (100%, *n* = 2)	–
ANCA	14 (8.97%, *n* = 156)**	13 (8.84%, *n* = 147)	1 (11.11%, *n* = 9)	0.581
CCP antibodies	36 (34.95%, *n* = 103)**	33 (34.02%, *n* = 97)	3 (50%, *n* = 6)	0.664
RF	41 (11.39%, *n* = 360)**	36 (10.78%, *n* = 334)	5 (19.23%, *n* = 26)	0.198
Clinical serum indexes				
RBC (10^12^/L)	4.42 (4.13, 4.77)	4.44 (4.14, 4.78)	4.29 (3.89, 4.57)	0.083
WBC (10^9^/L)	6.06 (4.82, 7.62)	6.11 (4.89, 7.62)	5.69 (4.22, 7.54)	0.171
HGB (g/L)	132.00 (120.00, 140.00)	132.00 (121.00, 141.00)	130.00 (113.00, 137.00)	0.194
PLT (10^9^/L)	208.48 ± 79.01	207.58 ± 77.76	220.08 ± 94.91	0.447
TP (g/L)	72.05 (66.88, 75.60)	72.20 (67.20, 75.60)	68.70 (62.65, 74.40)	0.122
ALB (g/L)	45.00 (41.40, 47.83)	45.10 (41.75, 47.90)	43.10 (35.65, 47.20)	0.054
AST (IU/L)	21.00 (17.00, 28.00)	21.00 (17.00, 28.00)	23.00 (17.00, 29.50)	0.272
ALP (IU/L)	74.00 (58.00. 96.00)	74.00 (59.00, 97.00)	72.00 (51.50, 90.50)	0.452
GGT (IU/L)	19.00 (12.00, 32.00)	19.00 (12.00, 30.50)	28.00 (14.50, 62.50)	0.049
CREA (umol/L)	60.00 (52.00, 72.50)	60.00 (52.00, 73.00)	57.00 (50.00, 68.50)	0.376
Cys‐C (mg/L)	0.83 (0.73, 0.95)	0.83 (0.73, 0.95)	0.85 (0.73,1.03)	0.481
IgM (mg/L)	1205.00 (835.50, 1690.00)	1210.00 (834.00, 1690.00)	1140.00 (800.00, 1765.00)	0.897
C3 (g/L)	0.86 ± 0.18	0.86 ± 0.18	0.84 ± 0.19	0.533
C4 (g/L)	0.22 ± 0.07	0.22 ± 0.07	0.21 ± 0.08	0.513

* 373 patients had definite diagnosis.

** 436 patients underwent extractable nuclear antigen (ENA) antibodies test, 381 patients underwent anti‐double‐stranded DNA (anti‐dsDNA) test, 60 patients underwent antikeratin antibodies (AKA) test, 156 patients underwent anti‐neutrophil cytoplasmic antibodies (ANCA) test, 103 patients underwent anti‐cyclic citrullinated peptide (CCP) antibodies test, and 360 patients underwent rheumatoid factor (RF) test.

## 4 DISCUSSION

As one of the most commonly seen IIF patterns in routine diagnostic laboratories performing ANA test on HEp‐2 substrates,^16^ the DFS pattern was initially identified in a patient with interstitial cystitis, but later in various disease conditions.^16^ Anti‐DFS70 antibodies target the lens epithelium‐derived growth factor (LEDGF) and react with conserved and conformational epitopes.^6^ There is evidence to suggest that DFS70/LEDGF plays a role in cell survival and in the protection against environmental stress.^17^ However, exploration of the exact clinical significance of the DFS pattern/anti‐DFS70 antibodies is still in progress. This study conducted systematic research on the prevalence and possible clinical association of the DFS pattern in a large teaching hospital and enriched information on medical practice.

In this study, a total of 36,869 patients who had positive ANA IIF test results were enrolled, and among them, patients with the DFS pattern accounted for 1.27%. Similar result (1.7%) was found in a study that included patients from 30 hospitals and 14 provinces across China.^18^ By comparison, a study from Australia showed a higher frequency (5.7%),^19^ which could be explained by differences in prevalence of the DFS pattern, races, etc. Females accounted for 76.97% of patients with DFS patterns, that is, 3.34 times as males in our study, consistent with a study in America (female‐to‐male ratio, 3.72).^4^ The average age of patients with the DFS pattern was 43.45 years, also similar to previous studies.^4^, ^19^ Of interest, our study identified 23.67% DFS pattern titers above/equal to 1:320, which seemed to be contrary to current reports that moderate to high titers were more common. Unfortunately, we are not able to give a reasonable explanation for the difference, thus more exploration is required.

In our study, we investigated the relationship of the DFS pattern with diseases and it turned out that inflammatory or infectious diseases, mental diseases, and SARDs ranked in the top three. As the target autoantibodies to the DFS pattern, anti‐DFS70 antibodies could be considered as “sensors” of microenvironmental stressors associated with inflammation, tissue damage, and altered expression of the DFS70 protein.^17^ This could explain the connection between the DFS pattern and a wide spectrum of clinical conditions to a certain extent. The proportion of patients diagnosed with SARDs was 18.23% of all patients with the DFS pattern, close to a study in America (20.47%),^4^ but higher than a study from Korea (8.23%),^20^ which might be due to differences in the definition of SARDs, prevalence of SARDs, sample size, etc.

Previous studies have inconsistent conclusions regarding the DFS pattern/anti‐DFS70 positivity as exclusion biomarker in SARDs. Some authors suggested that the isolated DFS pattern/anti‐DFS70 positivity could be used as exclusion biomarker.^6^, ^12^, ^21^ Their evidence mainly came from cohort studies, in which the DFS pattern/anti‐DFS70 positivity was more likely to appear in healthy individuals rather than SARDs patients, and was often accompanied by ENA antibodies, even if in SARDs patients. In addition, a 4‐year follow‐up study found that none of the DFS‐positive patients develop autoimmune diseases,^12^ constituting a crucial evidence to establish the DFS as exclusion biomarker in SARDs.^22^ Nevertheless, other authors argued that the DFS pattern/anti‐DFS70 positivity could not be responsible for excluding the diagnosis of SARDs. They discovered that their data did not support that anti‐DFS70 could exclude SARDs,^5^ and the DFS pattern was the only positive implication of autoimmune background for part of SARDs patients, and there was a 60% chance that these patients would be excluded from the diagnosis of SARDs according to the published prevalence of anti‐DFS70 antibodies in the DFS pattern.^22^ They also pointed out that it would take longer period than 4 years to confirm the correlation between DFS and autoimmune diseases, in response to that 4‐year follow‐up study.^22^ In our study, we identified 68 patients with SARDs. If the DFS pattern was used as exclusion biomarker of SARDs, these 68 patients would be missed. Besides, we did find that patients with positive ENA antibodies test results had a higher risk of suffering from SARDs, but through further analysis we found out that 47 patients who suffered from SARDs (and were not limited to RA or SLE) had negative antibodies test results and would be missed if isolated DFS pattern was considered as exclusion criterion of SARDs.

Based on literature review and results of this study, we consider it unwise to exclude the diagnosis of SARDs only depending on the appearance of the DFS pattern/anti‐DFS70 positivity (with or without positive ENA antibodies test results) for the time being. Reasons are as follows: (1) Missed diagnosis caused by using the DFS pattern/anti‐DFS70 positivity as exclusion criterion does exist. (2) Autoantibodies appearing in the serum may precede the clinical onset of SARDs by many years,^23^ thus it will take longer period to confirm. (3) The conclusion that the DFS pattern/anti‐DFS70 positivity can be used as exclusion criterion almost comes from observational retrospective studies that do not verify the diagnostic efficacy of this exclusion criterion. (4) Accurately identifying the DFS‐IIF pattern is not an easy task^24^ and may be interfered by the “pseudo‐DFS pattern.” The diagnostic performance of anti‐DFS70 antibody tests varies widely and has not been fully evaluated.^20^ Hence, in order to avoid missed or delayed diagnosis, as well as the physical and psychological harm caused to patients, it is unwise to exclude the diagnosis of SARDs only depending on the appearance of the DFS pattern/anti‐DFS70 positivity. Other related antibodies (such as ENA antibodies), clinical information of patients (such as symptoms and signs), and long‐term follow‐up are also of great importance.

The study suffers from several limitations. First, we did not carry out anti‐DFS70 antibodies test, so the prevalence and clinical association of antibodies were not shown in the study. We also did not take into account other unknown antibodies that might cause DFS pattern and “pseudo‐DFS pattern,” which had not been accepted widely. Second, as a single‐center study, results in this study have some limitations. Third, given our study a preliminary study, further exploration for the clinical significance of the DFS pattern is required.

In conclusion, we reported here the prevalence and possible clinical association of the DFS pattern in a large teaching hospital in China. A total of 469 patients revealed the DFS pattern, of which 92.96% had isolated DFS pattern and 23.67% had titers above/equal to 1:320. The average age of patients with the DFS pattern was 43.45 years, and females accounted for 76.97% of them. Ten different kinds of diseases made up the vast majority of the disease spectrum, in which inflammatory or infectious diseases (46.11%), mental diseases (21.45%), and SARDs (18.23%) ranked in the top three. The most common SARDs were RA, UCTD, and SLE. Forty‐six patients had positive or suspicious ENA antibodies test results. Patients with positive/suspicious ENA antibodies test results had a higher risk of suffering from SARDs; however, not all SARDs patients with DFS patterns had positive/suspicious ENA antibodies test results. If the DFS pattern with negative ENA antibodies test result was considered as exclusion criterion of SARDs, 47 patients would be missed. Hence, other related antibodies, clinical information of patients, and long‐term follow‐up are also of great importance to avoid missed or delayed SARDs diagnosis.

## CONFLICT OF INTERESTS

The authors declared no potential conflicts of interest with respect to the research, authorship, and/or publication of this article.

## AUTHOR CONTRIBUTIONS

Keyi Zhang, Chaojun Hu, and Bin Yang conceived the study concept and design. Zhenzhen Su and Jing Hu collected data. Zhuochun Huang performed statistical analysis. Keyi Zhang drafted the manuscript, and all authors significantly contributed to the revision of the manuscript and approved the submission.

## Data Availability

The data that support the findings of this study are available on request from the corresponding author. The data are not publicly available due to privacy or ethical restrictions.
